# Predictive factors influencing pregnancy rates after intrauterine insemination

**Published:** 2013-03

**Authors:** Arzu Yavuz, Oya Demirci, Hamdullah Sözen, Mehmet Uludoğan

**Affiliations:** *Department of Obstetrics and Gynecology, Zeynep Kamil Gynecologic and Pediatric Training Research Hospital, 34668, İstanbul, Turkey*

**Keywords:** *Intrauterine insemination*, *Pregnancy rate*, *Predictive factor*, *Gonadotropin*, *Progressive motile spermatozoa*

## Abstract

**Background: **So far, many studies investigated factors that affect pregnancy rates after intrauterine insemination (IUI). Various investigators have not agreed on the nature and ranking of these criteria.

**Objective:** The aim of this study was to assess the predictive factors for pregnancy rate after controlled ovarian hyperstimulation (COH)/ IUI.

**Materials and Methods:** Retrospective study of all patients undergoing IUI at Zeynep Kamil Gynecologic and Pediatric Training and Research Hospital from January 2006 to December 2009. In total 980 IUI cycles in 569 couples were analyzed. All women in the study underwent ovarian stimulation using gonadotropin and IUI was performed 36 h after triggering ovulation. The primary outcome measure was clinical pregnancy rates. Predictive factors evaluated were female age, body mass index (BMI), duration of infertility, type of infertility, follicle stimulating hormone (FSH) level and estradiol (E_2_) on third day of the cycle, number of preovulatory follicles, endometrial thichness, total motil sperm (TMS) count, and ratio of progressive motile sperm.

**Results: **The overall clinical pregnancy rate was 4.7%. Among the predictive factors after multivariate logistic regression analysis level of BMI (<25 kg/m²), number of preovulatory follicles (≥2), level of FSH (<9.4 IU/L), level of E2 (<80 pg/ml) and the ratio of progressive motile sperm (>50%) significantly influenced the clinical pregnancy rate.

**Conclusion:** Level of BMI, FSH, estradiol, number of preovulatory follicles and the ratio of progressive motile sperm may determine IUI procedure as optimum treatment model.

## Introduction

Intrauterine insemination (IUI) is an assisted reproduction procedure that places sperm directly into the uterus. Controlled ovarian hyperstimulation (COH) together with IUI is commonly offered to couples with subfertility factors not involving the fallopian tubes. IUI gained its popularity because it is simple, non-invasive, and a cost-effective technique (1). This method is indicated in cases of cervical infertility, relative male factor infertility, anovulation, endometriosis with a healthy fallopian tube, and unexplained infertility. 

Pregnancy rate after IUI differ between studies according to patient selection criteria, the presence of various infertility factors, ovarian stimulation methods, number of cycles performed, different sperm parameters and preparation technique (2). Although many factors have been reported as influencing pregnancy rates after IUI, the various investigators have not agreed the predictive factors for successful ongoing pregnancy (3-10). The purpose of our study therefore was to help clinicians predict IUI outcomes based on factors that can be assessed patients evaluations.

## Materials and methods

In this retrospective cohort study, we reviewed the medical records of women who underwent IUI at Zeynep Kamil Gynecologic and Pediatric Training and Research Hospital between January 2006 and December 2009. In total 980 IUI cycles in 569 couples with unexplained and male-factor subfertility were analyzed. The institution’s ethics committee approved the study and informed consent was obtained from the participants.

The study couples had at least one year infertility and had undergone a basic infertility evaluation consisting of detailed anamnesis (age ,duration of infertility, type of infertility, medical history, previous surgical interventions, lifestyles, body mass index ), hormone concentrations at the third day of menstrual cycle (FSH, LH, estradiol-17, TSH and PRL) and semen analysis. Physical and gynecological examinations were performed on all the women subjects. Tubal patency was confirmed by hysterosalpingography or laparoscopy. Laparoscopy or hysteroscopy was done when there was a possibility of pelvic adhesions, endometriosis, intracavitary lesions in uterus of the hysterosalpingography or transvaginal ultrasound. Patients with stage 3-4 endometriosis, intracaviter lesion in uterus, previous surgery on ovaries, and at least one closed tube were excluded from the study.

Body mass index (BMI) was calculated by dividing weight (kg) by square of height (m²) and patients were categorized into two groups as follows <25 BMI, and ≥25 BMI. Regarding the age of women, patients were divided into three groups as follows: <30 years old, 30-35 years old, and >35 years old. Duration of infertility was categorized as <6 years and >6 years. Ovarian function was estimated in terms of the serum concentrations of FSH and E_2_ before beginning of the cycle. The generally admitted thresholds are 9,4IU/L and 80 pg/ml, respectively (3). Patients were divided into 2 groups according to each threshold of FSH and E_2_. At the hCG administration day, 8 mm of endometrial thickness was admitted as cut off value and patients were divided into two groups as the ones with length of endometrium below 8 mm and the ones with this above 8mm.

All women in the study underwent ovarian stimulation using gonadotrophin (Gonal-F Ares-Serono, Geneva, Switzerland; Puregon, Organon International Inc, Roseland, NJ or hMG Menopur, Ferring SAS, St. Prex, Switzerland). Ovarian stimulation was initiated on the third day of the cycle with a dose of 75 IU. Initial dose of gonadotrophin was modified according to previous treatment, the duration of infertility, the woman’s hormone profile, level of body mass index and age. Initial dose of gonadotrophin was maintained throughout the first five days of stimulation. Necessary dose of gonadotrophin was altered after the first five days of stimulation depending on ovarian responses.

Ovarian and endometrial responses were monitored by transvaginal ultrasonography on the sixth day of stimulation and repeated after 2 or 3 days, depending on the follicular growth. When at least one follicle reached to 16 mm, 5000-10000 IU of hCG (Pregnyl N.V Organon) or 250 µg of recombinant hCG (Ovitrelle, Ares-Serono) was administered. Standard IUI was performed 36 hours after administration of hCG. The administration of hCG was withheld, and IUI was canceled in the stimulation protocols when more than three follicles with a diameter of at least 16mm or more than four follicles with diameter of at least 14 mm were present.

All the semen samples were collected by masturbation in the laboratory after three days of sexual abstinence and prepared less than 2 hours before insemination. After complete liquefaction for 30 minutes at room temperature, each sample was analyzed using World Health Organization/ Kruger guidelines. Semen preparation for IUI was performed with the density gradient centrifugation technique (20 minutes at 1300 rpm) and a washing step (6 minutes at 2200 rpm) with 2-ml culture medium (Quinn’s R sperm washing medium HTF with 5.0 mg/ml human albumin In vitro fertilization, inc. U.S.A.).

Bivalve speculum was used to expose the cervix and the cervical mucus was cleaned. Insemination catheter (Ainseblue-R RI.MOS, Mirandola Italy) was placed into the uterus approximately 0.5 cm below the fundus, 0.5 ml sperm preparation was injected slowly. A two weeks course of daily treatment with 90 mg micronized progesterone vaginal gel (Crinone 8%; Serono) was prescribed for luteal support. After this time, the woman was allowed to perform a pregnancy test (a serum β-hCG assay). Clinical pregnancies were defined as gestational sac in uterus.

**Statistical analysis**

Results were given as mean±SD deviation. Statistical analysis were performed by NCSS 2007 and PASS 2008 statistical software using Chi square test. Logistic regression analysis was used to investigate the existence of any correlation between the variables and the occurrence of pregnancy. 0.05 was regarded as statistically significant.

## Discussion

In this study, the overall pregnancy rate was found as 4.7% in 980 cycles. In comparison to other studies our result was lower. If you look at the studies related to IUI procedure with ovarian stimulation performed by hMG or gonadotrophin; Grigoriou *et al* reported the pregnancy rate as 14.87%, Merviel *et al* reported the pregnancy rate as 14.7%, Wainer *et al* reported the pregnancy rate as 12.91%, Iberico *et al* reported the pregnancy rate as 9.2%, and Steures *et al* reported the pregnancy rate as 8.2% per cycle (3-7). 

Because of our low overall pregnancy rate, we attempt to discover predictive factors for pregnancy after intrauterine insemination procedure. For this purpose logistic regression analysis was carried out and five predictive factors were identified. Level of BMI (<25kg/m²), number of preovulatory follicles (≥2), level of FSH (<9.4IU/L), level of estradiol (<80pg/ml) and the ratio of progressive motile sperm (>50%) were taken to be independent predictors for pregnancy rates after IUI procedure. 

When we looked carefully the negative predictive factors for pregnancy rate that we determined logistic regression analysis were found high incidence in the cycles. BMI ≥25mg/ml was found in 45% of the cycles, progressive motile sperm was found lower than ≤50% at 52% of cycles and one preovulatory follicle was found in 56% of the cycles. So the lower pregnancy rate can be the result of these high incidence of negative predictive factors. 

Our study showed that increased body mass index has a negative impact on IUI success. Different studies were published about the relation between increased BMI and reproductive disorders. A few number of studies found no association between BMI and pregnancy rates (10, 11). However large number of the studies showed a linear decrease in assisted reproduction treatment success with increasing BMI. Possible cause of these poor results in obese patients was explained with the quality of oocytes, hostile intrauterine environment and changes in sensitivity to insulin and androgen (12-15). Further analysis at obese infertile population is needed to understand the reason of poor results at assisted reproduction treatment.

Ovarian reserves were evaluated by the hormone assays on the third day of cycle. Our study showed significantly different pregnancy rates according to whether the level of FSH was below or above 9,4IU/L (5.6% vs. 0.6%) and the level of E_2_ was below or above 80 pg/ml (5.3% vs. 0.7%). Merviel *et al* studied the predictive value of FSH and E2 levels and admitted the same thresholds with our study (4). In contrast to our study, they found no significant difference in pregnancy rates between the groups. Also Mullin *et al* evaluated these parameters (16). Thresholds of FSH and E_2_ were admitted as15 IU/L and 80 pg/ml respectively. They found no significant difference in the per couple pregnancy rates between groups. 

With respect to preovulatory follicles, our study showed that number of follicles was a good prognostic predictor of IUI outcome. Highest pregnancy rate (14.4%) was found in cycles with three preovulatory follicles .We observed that at least three preovulatory follicles lead to a 7.79 fold increase in pregnancy rate. In consistency with our result Iberico *et al* reported that best IUI success rate was achieved with two or three preovulatory follicles (>15mm) (6). 

Nuojua-Huttunen *et al* and Dickey *et al* also reported IUI success rate is found 2-3 times more with 3 or more dominant follicles than one follicle (>15mm) (8, 17). Multifollicular development may result in an increased number of fertiliable oocytes and a better quality of endometrium and luteal phase thereby improving fertilization and implantation rates. In the present study, sperm quality (total motile sperm count, ratio of progressive motile sperm) was assessed. However in univariate analysis total motile sperm count was associated with increased pregnancy rate, in multivariate analysis total motile sperm count was not found as predictive factor for pregnancy after IUI. 

But, regarding the sperm characteristics; progressive motile sperm percentage was found independent factor for pregnancy after IUI in multivariate analyses. Nevertheless, contrary to our study Miller *et al*, Van Voorhis *et al*, Kang *et al*, and Iberico *et al* reported that total motile sperm count were independent predictive factors for pregnancy after IUI treatment (6, 18-20). Also, Yousefi *et al* described that the number of motil spermatozoa are factor with the highest impact on pregnancy after IUI treatment in woman with infertility for over ten years (21). 

Miller *et al* and Van Voorhis declared that the highest pregnancy rate after IUI was seen in case of motile sperm count above 10million. On the other hand, Nuojua-Huttunen *et al* did not found total motile sperm count and progressive motility as a predictive of IUI success (8). 

In our study, we did not find any difference in pregnancies according to whether the endometrial thickness was low or more than 8 mm. In the literature there are many studies that investigate the impact of endometrial thickness on the IUI success. Similar with us, Kolibianakis *et al* (clomiphene citrate was used for ovulation induction) and Yaman *et al* (patients were evaluated after IVF treatment) found no relation between pregnancy rate and endometrial thickness. In contrast, Esmailzadeh *et al*, Kovacs *et al* and Noyes *et al *accepted endometrial thickness as a main predictor of assisted reproductive treatment success (9, 22-25). Recently Merviel *et al* could not classify endometrial thickness as a predictive factor of pregnancy (4).

We did not show any significant difference on the rate of clinical pregnancy according to the length of infertility below 6 years and above 6 years. Similarly, Goverde *et al* and Merviel *et al* did not find significant difference in pregnancies between the groups with length of infertility below 6 years and above 6 years (4, 26). Contrary, Nuojua-Huttunen *et al*, Iberico *et al *and Erdem *et al* reported the duration of infertility as a negative effector of assisted reproductive treatment (6, 8, 27).

Age related decline in female fecundity has been reported in the past by Van Noord-Zaadstra *et al* and Kang *et al* (20, 28). These are the results of reduced uterine receptivity and decreased oocyte quality. The age has been accepted as a major predictive factor for pregnancy after IUI insemination by many authors (4, 7, 8, 26, 29). In our study pregnancy rates were found as 7.3% for under 30 years old, 3.5% for between 30-34 years old and 1.7% for over 35 years old. Nonetheless, statistical significance was found between the groups in univariate analysis, it was not found to be an independent factor for predicting pregnancies after IUI treatment. Similar with us, İberico *et al*, Mathieu *et al*, Khalil *et al* and Brzechffa *et al* declared that there was no negative effect on IUI success with advanced age (6, 30-32).

In our study, the infertile couples were investigated in short time interval. This short time interval is stronghold of our study. Unlike long prolonged studies, no changing has been made in the administration of IUI procedure, and all IUI procedures have been administered in a uniform manner by a unique team. The other stronghold of our study was that we excluded the women that have even though one-sided patent fallopian tube. Also, we excluded the women with stage 3-4 endometriosis and previous surgery on ovaries that are influencing ovarian response.

In conclusion, our study demonstrates that IUI procedure may be an optimum treatment model in some circumstances. Favorable patient characteristics for IUI treatment are defined as level of BMI<25 kg/m², level of E_2_ <80 pg/ml, level of FSH <9.4 IU/L, ratio of progressive motile sperm >50%, and the number of preovulatory follicle ≥2. We found lower clinical pregnancy rate compared with literature. We could attribute this conclusion to the high incidence of negative predictor factors. 
